# Are the Pathological Characteristics of Prostate Cancer More Aggressive or More Indolent Depending upon the Patient Age?

**DOI:** 10.1155/2017/1438027

**Published:** 2017-02-07

**Authors:** Guangjie Ji, Cong Huang, Gang Song, Gengyan Xiong, Dong Fang, He Wang, Han Hao, Lin Cai, Qun He, Zhisong He, Liqun Zhou

**Affiliations:** ^1^Department of Urology, Peking University First Hospital, Institute of Urology, Peking University, National Urological Cancer Center of China, Beijing, China; ^2^Department of Andrology, Peking University First Hospital, Institute of Urology, Peking University, National Urological Cancer Center of China, Beijing, China; ^3^Department of Radiology, Peking University First Hospital, Beijing, China

## Abstract

*Purpose*. To identify pathological characteristics of prostate cancer according to patient age at diagnosis.* Methods*. A retrospective review of 2,929 men diagnosed with prostate cancer was performed. Pathological characteristics were compared across age groups: ≤55, 56–75, and >75 years.* Results*. The study cohort included 133 patients (4.5%), 2,033 patients (69.5%), and 763 patients (26.0%) in the three age groups, respectively. The median pathological Gleason sums in the three age groups were 8, 7, and 8, respectively. The Gleason sum, primary Gleason score, and second primary Gleason score were significantly different among the three age groups (*Z* = 12.975, *p* = 0.002; *Z* = 9.264, *p* = 0.010; *Z* = 6.692, *p* = 0.035, resp.). The percentages of Gleason pattern 5 tumors for the three age groups were 44.4%, 32.3%, and 36.8%, respectively; they were significantly different (*χ*^2^ = 11.641, *p* = 0.003). The percentages of tumors with Gleason score grade groups 3–5 for the three age groups were 66.9%, 60.5%, and 66.3%, respectively; they were significantly different (*χ*^2^ = 9.401, *p* = 0.009).* Conclusions*. The present study indicated that men aged ≤55 years or >75 years show higher levels of clinically significant prostate cancer compared to patients between the ages of 55 and 75 years. Younger and more elderly male patients are more likely to have a more aggressive disease.

## 1. Introduction

Prostate cancer is considered a disease of older men and is infrequently reported in patients aged 55 years or younger [1]. However, presently over 10% of new cases of prostate cancer in the US occur in men aged 55 years or younger [2]. Compared with those in older men, the pathological characteristics of prostate cancer in patients 55 years or younger appear to be significantly different [3]. However, limited information is currently available on the pathological features of prostate cancer in younger men. Radical prostatectomy is recommended as the standard treatment modality for early stage prostate cancer in men aged 75 years or younger with a life expectancy of more than 10 years [4, 5]. However, patients over the age of 75 years with prostate cancer are more likely to receive treatment recommendations of primary hormonal therapy [6]. In addition, the pathological features of prostate cancer in elderly patients are different from those of other age cohorts.

It is widely accepted that prostate cancer comprises aggressive and indolent varieties. Indolent prostate cancer may exist for a long period without causing any symptoms or death. In contrast, aggressive prostate cancer may cause symptoms and lead to cancer-specific mortality. However, there is no consensus regarding the indolent or aggressive pathological characteristics of prostate cancer in younger or elderly patients with prostate cancer. To our knowledge, there is a lack of research reports regarding the main clinical and pathological characteristics of prostate cancer among different age groups. Thus, the aim of this retrospective study was to ascertain the differences in prostate cancer among different age groups, improve the accuracy of clinical diagnosis, and assist in treatment decisions.

## 2. Materials and Methods

### 2.1. Study Population and Design

A retrospective review of the pathological features of patients diagnosed with prostate cancer in the Department of Urology, Peking University First Hospital (Institute of Urology, Peking University, National Urological Cancer Center of China) from January 2001 to June 2016 was performed. All patients were pathologically diagnosed with prostate cancer via prostate biopsy and have not received any form of hormonal therapy or radiotherapy before biopsy. Accordingly, all the Gleason score information of patients was obtained from biopsy specimen. The ethics committee of the Peking University First Hospital approved this study.

Patients were stratified by age at the diagnosis into the following groups: ≤55 years (Group 1, young men), 56–75 years (Group 2, middle-aged and old men), and >75 years (Group 3, very old men). Pathological characteristics (Gleason sum, primary Gleason score, second primary Gleason score, and percent of Gleason pattern 5) were compared among the three groups. A new grading system, proposed by the International Society of Urological Pathology (ISUP) in 2014, has been incorporated in the new 2016 World Health Organization (WHO) prostate cancer reporting guidelines. The pathological characteristics of prostate biopsy can be classified into five distinct grade groups on the basis of the new grading system as follows: grade group 1 = Gleason score ≤ 6; grade group 2 = Gleason score 3 + 4 = 7; grade group 3 = Gleason score 4 + 3 = 7; grade group 4 = Gleason score 4 + 4 = 8; and grade group 5 = Gleason scores 9 and 10. Clinically significant prostate cancer is defined as grade groups 3–5. Comparisons were also made in the present study among the three age groups in the proportions of grade group 1-2 and grade group 3–5 tumors according to the newest grading system.

### 2.2. Statistical Analysis

All analyses were codified and performed using SPSS version 13.0 (SPSS Inc., Chicago, IL, USA). Pathological features were compared across age groups using the Kruskal-Wallis test, and statistical significance was set at a *p* value < 0.05. ANOVA tests were applied to analyze the difference of median age between each of the Gleason grade groups. Further comparisons (Group 1 versus Group 2; Group 2 versus Group 3) were performed using the Mann-Whitney-Wilcoxon test and the significance level was set at *p* < 0.025. Pearson's chi-square test was applied to compare the percentage of Gleason pattern 5 and the percentage of Gleason score grade groups 3–5 among the age groups. A *p* value < 0.05 was considered significant.

## 3. Results and Discussion

### 3.1. Results

A total of 2,929 men were pathologically diagnosed with prostate cancer in our institution between January 2001 and June 2016. Of the 2,929 men evaluated, 133 (4.5%) were in Group 1 (≤55 years, young men); 2,033 (69.5%) were in Group 2 (56–75 years, middle-aged and old men); and 763 (26.0%) were in Group 3 (>75 years, very old men). More clinical information of all patients is shown in Table 1.

The median pathological Gleason sums were 8 (range: 6–10), 7 (range: 3–10), and 8 (range: 3–10) in Groups 1, 2, and 3, respectively. There were significant differences among the three age cohorts in pathological characteristics including Gleason sum, primary Gleason score, and second primary Gleason score (*p* < 0.05). After further comparisons performed between Groups 1 and 2 and Groups 2 and 3, it was found that Gleason sum, primary Gleason score, and second primary Gleason score were significantly higher in Group 3 than in Group 2 (*p* < 0.025). All data are presented in Table 2. Meanwhile, the median age was 71 years (range: 42–87), 70 years (range: 36–87), 71 years (range: 37–89), 72 years (range: 43–91), and 71 years (range: 33–89) in Gleason grade groups (GGG) 1, 2, 3, 4, and 5, respectively (*F* = 2.15, *p* = 0.072).

The percent of Gleason pattern 5 was significant different among the three groups (44.4%, 32.3%, and 36.8%, resp., *χ*^2^ = 11.641, *p* = 0.003, Table 3). When compared to Group 2 (56–75 years), Groups 1 (≤55 years) and 3 (>75 years) showed significantly higher percentages of Gleason pattern 5 (*χ*^2^ = 8.183, *p* = 0.004; *χ*^2^ = 5.065, *p* = 0.024, resp.).

**Figure 1:**
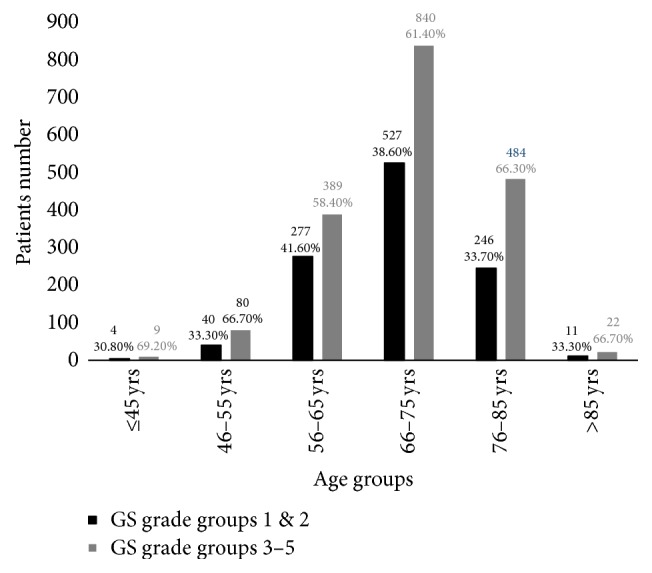
The distribution of Gleason score (GS) in different age groups.

The distribution of Gleason scores in different age quartiles (≤45, 46–55, 56–65, 66–75, 76–85, and >85 years), based on the new grading system proposed by the 2016 WHO prostate cancer reporting guidelines, is given in Figure 1. When a comparison was performed across the three age groups (≤55, 56–75, and >75 years) for all study subjects (Table 4), the percentages of patients assigned to grade groups 3–5 were higher than those assigned to grade groups 1-2 in all three age groups. There were statistically significant differences in the percentages of patients from each of the age groups assigned to Gleason score grade groups 3–5, with 66.9%, 60.5%, and 66.3% of patients in Groups 1, 2, and 3 (*χ*^2^ = 9.401, *p* = 0.009). The difference between patients in Groups 2 and 3 was also significant (*χ*^2^ = 8.103, *p* = 0.004), whereas no statistically significant difference was observed between Group 1 and Group 2 (*χ*^2^ = 2.190, *p* = 0.139).

### 3.2. Discussion

Prostate cancer is the most commonly diagnosed malignant tumor in older men, but it is infrequently reported in younger men [1]. Most previous studies on prostate cancer have led many clinicians to reach a consensus that elderly men are not good candidates for radical prostatectomy and they would present better outcomes in response to hormonal therapy [4, 5]. However, till date, there is no specific criterion for defining the different age groups of prostate cancer [1]. An earlier retrospective study conducted on young patients discussed the clinicopathological features of prostate cancer in men under 50 years of age [7]; however, there have also been several reports classifying adults under 55 or 59 years, respectively, as young patients [1, 8]. A retrospective report focusing on age-related outcomes for elderly men with prostate cancer used a cutoff age of 70 years [9]. Moreover, a large body of literature on the oncological outcomes of prostate cancer has suggested that patients aged more than 75 years should not be treated with radical prostatectomy owing to their very short life expectancy [10]. In the current study, we assigned 2,929 patients with prostate cancer into three age groups: Group 1 (≤55 years, young men), Group 2 (56–75 years, middle-aged and old men), and Group 3 (>75 years, very old men). The purpose of the present study was to identify and analyze the pathological characteristics of prostate cancer in different age groups.

Several reports have indicated that older men often harbor more advanced tumors [11–13]. Our findings suggested a significant difference in Gleason sum among the three age groups (scores of 8, 7, and 8 in Groups 1, 2, and 3, resp.). There was also a significant difference when Groups 2 and 3 were compared in isolation. These results indicated that patients aged more than 75 years are more likely to be diagnosed with high-risk prostate cancer. However, a recent study focusing on Korean patients found that radical therapy might be an appropriate treatment option for selected healthy men aged 75 years or more [14]. Although the differences between the Gleason sum in Groups 1 and 2 was not statistically significant (*p* = 0.034 [>0.025]), this finding might have been observed because of the large imbalance in the patient population, in which only 133 subjects were ≤55 years of age and there were 2,033 patients between the ages of 55 to 75 years. The results indicated a trend towards the association of patients aged ≤55 years with higher biopsy Gleason scores compared to the middle-aged and old patient group.

Most researchers have concluded that young patients with prostate cancer have less aggressive clinicopathological characteristics and more favorable outcomes compared with older men [15–17]. The Cancer of the Prostate Risk Assessment (CAPRA) score, a widely used predictive model for biochemical recurrence and survival after radical prostatectomy, indicates that age under 50 years is one of the independent favorable risk factors [18]. Kinnear et al. [16] argued that Australian men aged ≤50 years diagnosed with prostate cancer have more favorable pathological features. Similarly, two other studies reported that early age at diagnosis was associated with less advanced disease characteristics and improved outcomes [9, 19]. Nevertheless, several studies showed completely different perspectives, detecting a poor prognosis in younger patients [7, 20].

A recent study conducted to analyze the prognostic significance of the percent of Gleason pattern 4 suggested that an increase in the percent of Gleason pattern 4 correlated with adverse risk and poorer outcomes [21]. Many clinicians believe that the Gleason pattern 5 might also predict an adverse prognosis in prostatic neoplasms. Our findings showed that both the young and the very old group had significantly higher percentages of Gleason pattern 5 than the middle-aged and old group, which indicated that the patients younger than 55 years or older than 75 years in this cohort appeared to have a greater likelihood of tumors with aggressive behavior. The new grading system, adopted by the new 2016 WHO prostate cancer reporting guidelines, was shown to provide a stratification instrument for tumors that is more accurate in predicting progression than the Gleason risk stratification system (≤6, 7, and 8 to 10) [22]. One large multi-institutional study [23] revealed that the patients diagnosed with grade group 1 tumors (Gleason score ≤ 6) did not appear to experience metastasis to lymph nodes, with a more predictable and favorable prognosis. Grade group 2 (Gleason score 3 + 4 = 7) also has a relatively favorable prognosis, with rare metastases. Comparing the percentage of grade groups 3–5 between all three age groups, we found that the percentage in the very old group was statistically higher than that in the middle-aged and old group, while there was no significant difference between the percentage in the young group and the middle-aged and old group. Given the higher percent of Gleason pattern 5, there might be fewer cases of Gleason scores 4 + 3 and 4 + 4 in the young group. The results suggested that the younger and older age at the time of prostate cancer diagnosis were associated with aggressive cancer characteristics.

The results of our research were contrasting to the findings of most published reports, which concluded that younger men have better disease-free outcomes compared to older patients [24]. One reason for this finding might be the different grouping strategy [16]. It may also be due to the ethnic diversity among the studies. The incidence of prostate cancer in younger men had increased remarkably since the initiation of widespread use of serum prostate specific antigen (PSA) screening; however, the results of PSA screening would be affected by individual differences in malignant latency [3]. The slow-growing or indolent tumors would have a better opportunity to be identified, while missing the timely diagnosis of early onset prostate cancer (diagnosis at ≤55 years) because of the very short window for detection before symptoms appear. Consequently, it is no accident that younger patients diagnosed with early onset prostate cancer would tend to have more advanced disease characteristics and higher cancer-specific mortality than other subgroups. At present, there remains a lack of large studies on the clinicopathological features of prostate cancer in Chinese patients who were diagnosed with the disease at an early age. In addition, a prior report indicated that race might play a significant role in the tumor biology of prostate cancer in younger adults [9]. In the present study, the existing data suggested that early onset prostate cancer occurred in a higher proportion in the Chinese younger population.

Many studies have demonstrated that men with a family history or genetic mutations were at increased risk of prostate cancer, particularly at a young age. Edwards et al. [25] argued that the risk of prostate cancer was almost 23-fold higher in* BRCA2* mutation carriers compared to those with no mutation. Moreover, Sigurdsson et al. [26] found that* BRCA2* mutation in the Icelandic population might be a possible biomarker for an aggressive form of prostate cancer. Two other reports also confirmed that* BRCA2* mutations were associated with more advanced disease and shorter disease-specific life expectancy [27, 28]. Furthermore, a novel gene variant named* HOXB13 G84E* was identified by several genetic studies that found a strong relationship between this mutation and susceptibility to prostate cancer. However, interestingly, patients with* HOXB13 G84E* germline mutation appeared to have a more favorable prognosis [29–31]. These observations might open up a new avenue for the screening and diagnosis of the selected germline mutations and even point to new targets for cancer therapy.

There are two reasons why our study included merely pathological grading of prostate cancer in this cohort, without involving the clinical or pathological stages. Firstly, the tumor staging could not characterize the pathological features well because the results might have been affected by the method or timing of diagnosis. Secondly, the urologists could only determine exact pathological stages of diseases in the patients who underwent prostatectomy. Thus, there were no analyses regarding the tumor stages in this study.

The present study has certain limitations and constraints, of which the most obvious is the deficiency of a retrospective approach. Another important limitation is that all the grade information of the patients was evaluated via biopsy not surgical specimens which could be more representative of prostate cancer progression compared to biopsy tissues, despite the fact that biopsy outcome may be more clinical instructive for urologist at tumor diagnosis moment.

## 4. Conclusions

The results of this single institution retrospective analysis indicated that in relation to differences in Gleason scores among various age groups, men aged ≤55 years or >75 years, show significantly higher percentages of Gleason pattern 5 compared to patients aged 56–75 years. Younger and elderly ages in this Chinese cohort are associated with more aggressive disease characteristics. Further studies that evaluate the clinicopathological features of prostate cancer in different age groups are warranted.

## Figures and Tables

**Table 1:** Clinical data of all 2929 patients in different age groups.

	Total (2929)	Group 1 (133)	Group 2 (2033)	Group 3 (763)
Median Age (years)	71 (33–91)	52 (33–55)	69 (56–75)	79 (76–91)
Median tPSA (*μ*g/dL)	19.0 (1.7–>1000)	20 (3.7–500)	18.0 (1.7–>1000)	20.7 (1.7–>1000)
Median BMI (kg/m^**2**^)	24.2 (15.1–41.7)	25.1 (18.0–32.5)	24.1 (16.4–40.1)	23.7 (15.1–41.7)
T stage				
T1-T2	1490 (50.8%)	56 (42.1%)	1128 (55.5%)	306 (40.1%)
T3-T4	1439 (49.2%)	77 (57.9%)	905 (44.5%)	457 (59.9%)
N				
0	2021 (68.9%)	90 (67.7%)	1450 (71.3%)	481 (63.0%)
1	908 (31.1%)	43 (32.3%)	583 (28.7%)	282 (37.0%)
M				
0	1976 (67.5%)	86 (64.7%)	1423 (70.0%)	467 (61.2%)
1	953 (32.5%)	47 (35.3%)	610 (30.0%)	296 (38.8%)

Group 1: age ≤ 55 years (young men).

Group 2: age 56–75 years (middle-aged and old men).

Group 3: age > 75 years (very old men).

tPSA: total prostate-specific antigen; BMI: body mass index.

**Table 2:** Comparisons of pathological characteristics between different groups.

	Groups 1, 2, and 3		Group 1 versus Group 2		Group 2 versus Group 3	
	*Z*	*p* value	*Z*	*p* value	*Z*	*p* value
Gleason sum	12.975	0.002^*∗*^	2.120	0.034	3.155	0.002^*∗*^
Primary Gleason score	9.264	0.010^*∗*^	1.954	0.051	2.564	0.010^*∗*^
Second primary Gleason score	6.692	0.035^*∗*^	1.496	0.153	2.285	0.022^*∗*^

Group 1: age ≤ 55 years (young men).

Group 2: age 56–75 years (middle-aged and old men).

Group 3: age > 75 years (very old men).

^*∗*^Statistically significant difference.

**Table 3:** The percentages of Gleason pattern 5 tumors in the three age groups.

	Group 1 (≤55 years)	Group 2 (56–75 years)	Group 3 (>75 years)
Gleason pattern < 5	74 (55.6%)	1376 (67.7%)	482 (63.2%)
Gleason pattern = 5	59 (44.4%)	657 (32.3%)	281 (36.8%)

**Table 4:** The percentages of Gleason grade groups (GGG) in the three age groups.

	Group 1 (≤55 years)	Group 2 (56–75 years)	Group 3 (>75 years)
GGG 1	21 (15.8%)	305 (15.0%)	95 (12.5%)
GGG 2	23 (17.3%)	499 (24.5%)	162 (21.4%)
GGG 3	16 (12.0%)	308 (15.2%)	104 (13.6%)
GGG 4	19 (14.3%)	320 (15.7%)	145 (18.9%)
GGG 5	54 (40.6%)	601 (29.6%)	257 (33.7%)

GGG 1-2	44 (33.1%)	804 (39.5%)	257 (33.6%)
GGG 3–5	89 (66.9%)	1229 (60.5%)	506 (66.3%)

SUM	133 (100%)	2033 (100%)	763 (100%)
